# Targeting Neutrophil Function as Therapy for Hidradenitis Suppurativa

**DOI:** 10.3390/ijms27042076

**Published:** 2026-02-23

**Authors:** Eric Meldrum, John R. Ingram

**Affiliations:** 1 Citryll B.V., 5349 AB Oss, The Netherlands; 2 Division of Infection & Immunity, Cardiff University, Cardiff CF10 3AT, UK; ingramjr@cardiff.ac.uk

**Keywords:** hidradenitis suppurativa, neutrophil, inflammation, chemotaxis, eltrekibart, Dipeptidyl peptidase 1, Brensocatib, neutrophil extracellular traps, NETs, CIT-013

## Abstract

Hidradenitis suppurativa (HS) is a chronic, recurrent inflammatory skin disease characterized by painful nodules, abscesses, and epithelialized tunnels, predominantly affecting flexural regions. With a global prevalence of approximately 1%, HS has a significant negative impact on quality of life. Multi-omics and histopathology studies have revealed a complex interplay between innate and adaptive immunity in HS, with neutrophils emerging as important drivers of inflammation. While therapies targeting TNF-α and IL-17 isoforms offer a degree of benefit, significant unmet need remains. Neutrophil signatures in HS lesions and the circulation underscore the rationale for selective modulation of neutrophil function. Strategies advancing through clinical trials include inhibition of chemokine-mediated trafficking, neutrophil serine protease inactivation and suppression of neutrophil extracellular traps (NETs), which amplify inflammatory and autoimmune responses. These emerging therapies mark a significant shift toward targeted neutrophil modulation, offering new opportunities to improve outcomes for patients with HS.

## 1. Introduction

Hidradenitis suppurativa (HS) is a chronic inflammatory skin disease characterized by recurrent, painful skin lesions including inflammatory nodules and abscesses in the axillae, groin, and other flexural areas [1]. Irreversible tissue destruction and extensive scarring may result as the disease progresses. Disease onset is typically in early adulthood, soon after puberty and it remains unknown whether female menopause improves the condition [2]. HS is a common disease, affecting 1% of the global population [2,3] but is often underreported and misdiagnosed [4]. It can have a large impact on quality of life, with EQ5D scores similar to those for severe COPD and cerebral stroke [5].

In Europe and North America, the female to male ratio is 3:1, while the ratio is reversed in South Korea [6]. There is a strong association with cigarette smoking however, evidence is limited as to whether smoking cessation improves prognosis [2]. In Europe and North America, HS is associated with obesity and one study has found that disease severity increased in line with body mass index (BMI) increments [7]. HS is associated with a reduced life expectancy of 15 years in Finnish patients, with higher risks of cardiovascular disease and completed suicide [8]. HS is also linked with other chronic inflammatory conditions, in particular inflammatory bowel disease, seronegative arthritis and psoriasis [2]. There are also associations with depression, acne, polycystic ovary syndrome, obstructive sleep apnea and Down syndrome [2].

The primary pathogenic event in HS involves hair follicle occlusion due to hyperkeratosis and hyperplasia of the follicular epithelium, followed by follicular dilatation and eventual rupture to release commensal microbiota and keratin which trigger inflammatory cell infiltration [9]. This results in recurring, painful nodules and pus-containing abscesses which, in patients with moderate to severe disease, can progress into inflamed, pus-draining, inter-connected epithelialized skin tunnels (previously also known as sinus tracts or fistulae) that are characteristic of HS [10]. These skin tunnels cause substantial pain, are predictors of poor response to therapy [11] and are associated with a more aggressive disease course [12,13]. There have been several attempts using latent class analysis to identify different phenotypic subtypes of HS to help predict those whose disease will worsen quickly, also known as ‘rapid progressors.’ However, to date these have been unsuccessful [14,15]. Similarly, demographic and phenotypic information has not indicated likely response to different therapeutic modalities and personalized therapy will probably need integration of phenotype with immunophenotype and genetic data.

The multifactorial nature of the innate and adaptive immune drivers of HS has been established by histopathology studies showing immune cell infiltration (i.e., neutrophils, lymphocytes, macrophages, mast cells, and dendritic cells) into regions proximal to ruptured hair follicles [10,16,17,18]. This has been expanded upon by transcriptomic [19,20,21,22], proteomic [23], lipidomic [24] and multi-platform [10,25] studies on skin biopsies and blood. Such detailed understanding of the features of HS lesions has instructed the successful development of targeted therapies that improve HS patient symptoms [26,27,28]. However, therapies targeting TNF-α, IL17-A or both IL-17A and IL-17F bring modest relief in terms of HiSCR50 primary outcome attainment to participants in clinical trials. This reflects the heterogeneous nature of the disease and suggests additional pathogenic factors remain for therapeutic targeting. One common motif from histopathology and “omic” analyses, in keeping with HS being a neutrophilic dermatosis, has been the association of HS with neutrophil signatures in the inflamed skin and circulation of HS patients. HS therapeutics targeting neutrophils and their proinflammatory actions offer a differentiated approach that may build upon the modest patient benefit of approximately 20% placebo-corrected HiSCR50 response from the currently licensed targeted therapies.

The focus of this review will be on therapeutic approaches now in clinical development that directly target the contribution of neutrophils to the inflammation pathology of HS. Therapeutic approaches targeting additional immunological pathways have been comprehensively reviewed elsewhere [29].

### 1.1. The Neutrophil

**Figure 1:**
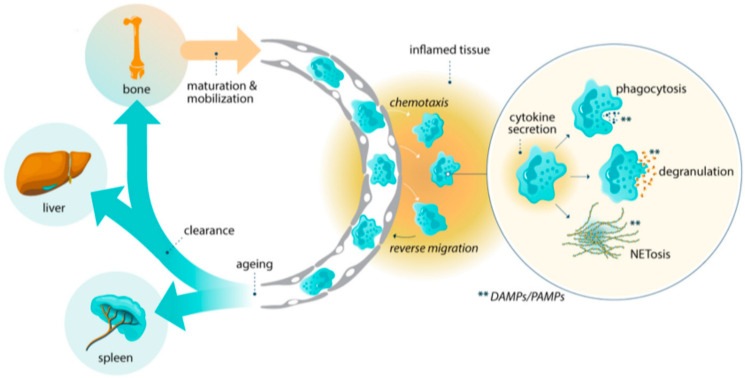
Neutrophils, derived from myeloid progenitors in the bone marrow, are sentinels of innate immunity. They survey the body for pathogen and damage associated molecular patterns (PAMPs and DAMPs) and rapidly migrate to sites of infection or inflammation to deploy a diverse set of specialized responses. Upon activation, neutrophils may secrete proinflammatory cytokines, internalize pathogens through phagocytosis for rapid destruction or release bactericidal proteins, reactive oxygen species (ROS), and pro-inflammatory mediators via degranulation. An additional response is a distinct cell death pathway which culminates in the rupture of the neutrophil and the formation of neutrophil extracellular traps (NETs). These are web-like structures of chromatin enriched with granule components, inflammatory mediators, and DAMPs that immobilize and kill pathogens.

Comprising approximately 50–70% of circulating leukocytes, neutrophils are the most abundant human white blood cell. Derived from myeloid progenitors in the bone marrow, and with a lifespan range of 7–90 h, neutrophils patrol the body for pathogen and damage associated molecular patterns (i.e., PAMPs and DAMPs, respectively) and rapidly migrate to inflamed and infected tissue offering a repertoire of different specialized responses to environmental threats. As the first line of innate immune defense, neutrophils also signal for recruitment of other immune cells to sites of infection and inflammation [30]. Migration into and reverse migration from inflamed tissue, context specific triggers and aging all contribute to divergent neutrophil phenotypes with distinct molecular signatures and specialized responses (Figure 1). Thus, the neutrophil is not a uniform cell type but a phenotypically plastic, heterogenous population of cells collectively capable of balanced responses in different innate immune settings. This heterogeneity is exemplified by low-density granulocytes (LDGs), so called because of their low buoyancy following density centrifugation. These were first described in the blood of patients with systemic lupus erythematosus (SLE) [31] and subsequently, increasing numbers were shown to correlate with higher juvenile-SLE disease activity [32]. While no unifying surface phenotype or marker yet defines this population, they exist in an activated state and have been observed in multiple inflammatory and autoimmune conditions [33]. While they are present in healthy individuals, data suggests that they increase in number and become inappropriately activated in the context of immune-mediated inflammatory diseases.

Given the essential contribution of neutrophils to innate immune defense, depletion therapeutic strategies raise tolerability concerns. Consequently, emerging therapies seek to attenuate neutrophil-mediated inflammatory damage without compromising core immune functions. Although selectively targeting pathogenic neutrophil subsets such as LDGs would be attractive, such approaches require a better understanding of disease-specific neutrophil surface markers and other phenotypic discriminators. Consequently, therapeutic approaches targeting the neutrophil in HS have focused on specific functions linked to disease activity. These strategies aim to reduce neutrophil-mediated inflammation by inhibiting their migration into the skin, inhibiting the activity of destructive neutrophil serine proteases (NSPs) or reducing levels of tissue neutrophil extracellular traps (NETs) to suppress proinflammatory responses they initiate and magnify.

### 1.2. ELR + CXC Chemokines as Therapeutic Targets in HS

Neutrophil migration from the circulation to sites of inflammation involves slow rolling, enhanced endothelial adhesion and intraluminal crawling prior to trans-endothelial migration and chemotaxis toward an increasing concentration gradient of chemoattractants within inflamed tissue [34]. The four classes of neutrophil chemoattractants in humans (chemokines, chemotactic lipids, complement anaphylatoxins and formyl peptides) all function by activating selective G-protein coupled receptors with chemokines differing from the others in that they have a degree of selectivity for leukocyte subsets. The chemokine CXCL8 (IL-8) is the most potent neutrophil attracting and activating chemokine and CXCR1 and CXCR2 are the predominant neutrophil receptors [35]. After unsuccessful efforts to antagonize CXCR1 and CXCR2 receptors with small molecules, the lead therapeutic approach in HS has become a monoclonal antibody that binds a shared epitope in all the CXC chemokines with a conserved N-terminal ELR amino acid motif (i.e., CXCL1 to 3 and CXCL5 to 8). This neutralizing, pan-ELR + CXC chemokine monoclonal antibody, eltrekibart (LY3041658), potently inhibits neutrophil migration [36] without affecting other neutrophil functions and has progressed into clinical development in HS.

In a randomized, double-blind, placebo-controlled phase 2 study of eltrekibart in moderate to severe HS patients (NCT04493502), patients received either 600 mg eltrekibart (*n* = 45) or placebo (*n* = 22) by IV administration every other week for 16 weeks. All study participants then progressed to an open-label extension period, receiving 600 mg IV eltrekibart every other week for 20 weeks. Treatment-emergent adverse events were mostly mild to moderate and at similar rates as placebo [37]. In this study, participants in the treatment group had a significantly greater mean reduction in the number of abscesses and inflammatory nodules at week 16 relative to placebo. Furthermore, 81% of the patients showing a HiSCR50 response at week 16 maintained their improvement at week 36. How these efficacy signals relate to the suppression of neutrophil infiltration has not been reported and uncertainty remains therefore, whether other parallel chemoattractants such as C5a or leukotrienes remain able to sustain significant neutrophil trafficking into the tissue. However, the outcome of this study represents an important first clinical validation of targeting the neutrophil as a novel therapeutic approach in HS. Eltrekibart has progressed to a 16-week, double blind, placebo-controlled, sub-cutaneous dose ranging study in moderate to severe HS patients (NCT06046729) which is anticipated to complete during the second half of 2026.

### 1.3. Dipeptidyl Peptidase 1 as a Therapeutic Target in HS

Another neutrophil targeting therapy in clinical development for HS is brensocatib, an oral reversible competitive inhibitor of Dipeptidyl peptidase 1 (DPP-1, also known as Cathepsin C). During neutrophil maturation in the bone marrow, DPP1 activates precursor neutrophil serine proteases (NSPs) by removing N-terminal dipeptides. This enables maturation of neutrophil elastase, cathepsin G, and proteinase 3 [38]. Stored in the azurophilic granules of neutrophils, these proteases provide significant anti-microbial protease activity upon pathogen phagocytosis or granule exocytosis. However, in the context of chronic neutrophilic inflammation, sustained extracellular activity of these proteases enhances tissue damage and inflammation through extracellular matrix degradation and by targeting cytokines, chemokines and a variety of cell surface receptors for activation [39].

Neutrophil-mediated inflammation is central to the cycle of inflammation, impaired mucociliary clearance, structural airway damage and recurrent infection that promotes progression of bronchiectasis [40]. Brensocatib has been shown to lower NSP activity in the sputum of bronchiectasis patients [41] to result in a clinically meaningful effect on the burden of pulmonary exacerbations [42] and in 2025, brensocatib (Brinsupri) was approved for once-daily, oral use for the treatment of bronchiectasis.

As part of an evaluation of brensocatib in other neutrophil-mediated diseases, a Ph2b clinical study in HS with treatment for at least 6 months is ongoing and scheduled for completion by mid-2026 (NCT06685835). The hope is that targeting neutrophil inflammation by this approach will improve HS patient outcomes as it has done in bronchiectasis.

### 1.4. Neutrophil Extracellular Traps as Therapeutic Targets in HS

**Figure 2:**
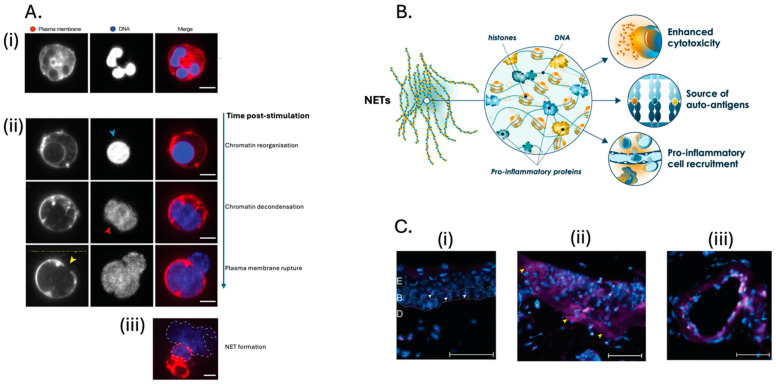
(**A**) A resting human neutrophil characterized by multi-lobed nucleus (i) stimulated to undergo NET formation (ii) undergoes deamination (i.e., citrullination) of histone arginine residues, chromatin reorganization, breaching of the nuclear membrane and dissolution of the granules to mix the chromatin with granule and cytosolic contents. Chromatin continues to decondense within the cytoplasm to provide the outward motive force for plasma membrane rupture and extracellular release of citrullinated chromatin in association with the granule contents and numerous potent DAMPs (iii) [45,46]. Scale bars in (i) and (ii) are 5 µm and in (iii) is 10 µm. (Adapted with permission from [47]. Copyright 2023, Citryll BV) (**B**) The heterogeneous composition of NETs means their pathogenic role is attributable to many different components acting in tandem (e.g., citrullinated histones, NSPs, myeloperoxidase, cytokines, chemokines and danger signals such as HSP70 and calprotectin). The aggregate effect of NETs is cytotoxicity to amplify local DAMP levels, enrichment of citrullinated autoantigens and endothelial activation to enhance chemotaxis of immune cells for further amplification the inflammatory state. (**C**) Immunohistochemical staining of unaffected skin (i) for citrullinated histone H3 (CitH3; pink) and DNA (blue) showed punctate intracellular CitH3 (white arrowheads) in the epidermis (E), basal layer (B) and dermis (D). HS lesional skin CitH3 staining (ii) shows a diffuse extracellular signal, indicating neutrophil extracellular traps (NETs; yellow arrowheads). Staining of an HS epithelialized skin tunnel (iii) also shows abundant NET positivity. (Adapted with permission from [48]. Copyright 2025, Citryll BV).

In 2004, Zychlinsky, Brinkmann and colleagues [43] identified neutrophil extracellular traps (NETs) as chromatin-based structures enriched with granule proteins that capture and kill extracellular bacteria. Dysregulated NET formation, even in the absence of infection, has since been implicated in the pathogenesis of numerous immune-mediated inflammatory diseases. (Figure 2A,B, [44]).

While an abundance of neutrophils in HS lesions had been previously reported by histopathology studies, Kaplan, Byrd and colleagues were the first to establish a positive correlation between HS lesion severity and increasing NETs levels in biopsies [49]. Additional biopsy studies have shown NETs are widely distributed across both lesional and perilesional regions of HS skin and are associated with HS structures such as the lining of epithelialized skin tunnels (Figure 2C, [45]). While HS is an inflammatory disorder of the skin, evidence of NET-associated dysregulation of innate and adaptive immunity has also been described in the blood. As reported for many chronic inflammatory diseases [50,51,52,53,54] a subset of neutrophils in the peripheral blood of HS patients display a significantly enhanced capacity for NET formation even in absence of exogenous stimuli [49]. Increased numbers of LDGs are a candidate neutrophil sub-type responsible for this activated phenotype. Markers of NETs and innate immune activation (e.g., citH3, nucleosomes and calprotectin) are also elevated in the serum of HS patients in a manner that positively correlates with disease severity [48]. Elevated NETs in HS serum may, at least in part, be due to an impaired ability to degrade NETs [55]. In the serum of most HS patients, this defect is reversed by the addition of exogenous nucleases, but in a sizeable proportion, anti-DNase auto-antibodies protect serum NETs from digestion. The autoimmune aspects of HS are an emerging research topic with important diagnostic and therapeutic implications. Evidence of disrupted self-tolerance includes autoantibodies directed against NET components and citrullinated proteins detected in patient serum [49,56]. This is consistent with findings in other immune-mediated inflammatory diseases, where excessive extracellular citrullinated autoantigens generated through NET formation contribute to adaptive immune dysregulation [57,58,59].

One final body of supporting evidence for NETs as pathogenic factors in HS comes from mechanistic studies on the NOTCH-γ-secretase signaling pathway. Several studies have shown that HS pathogenesis is associated with heterozygous mutations in different sub-units of the γ-secretase complex [60]. This complex mediates cleavage of multiple substrates including Notch, whose activation through proteolytic processing regulates gene expression. The discovery that NETs activate Notch–γ-secretase signaling to drive pro-inflammatory responses in macrophages and pro-fibrotic changes in dermal fibroblasts links NET abundance to key genetic associations in HS [61].

**Figure 3:**
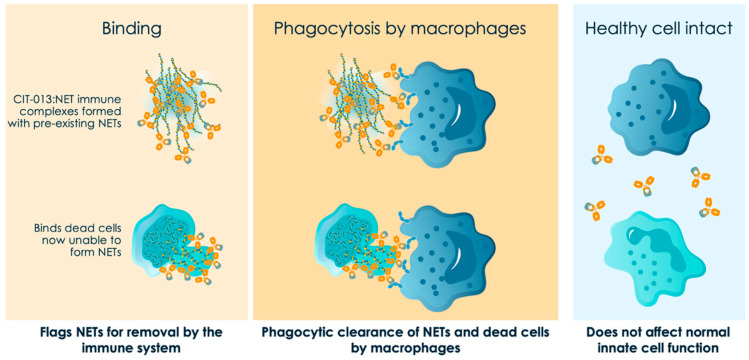
Citrullinated histones are unique to extracellular traps, thus allowing the anti-inflammatory action of CIT-013 through binding the citrullinated N-terminus of histones H2A and H4. In addition to binding pre-existing tissue NETs (upper row), CIT-013 targets citrullinated histones exposed at the cell surface during the final stage of NET formation, when the plasma membrane begins to lose integrity (lower row). This action prevents cell rupture and blocks the release of new NETs. Both types of the extracellular immune complexes formed are recognized and cleared by Fc-dependent phagocytosis [47]. Since extracellular citrullinated histones are present exclusively after NET formation, CIT-013 is highly specific and does not interfere with other neutrophil innate immune functions.

Strategies for therapeutic reversal of NET accumulation in inflamed tissue have focused upon two approaches: inhibition of Peptidylarginine Deiminases (PADs) that catalyze the citrullination necessary for chromatin decondensation, cell rupture and NET formation [62,63,64] and the targeting of specific citrullinated proteins with monoclonal antibodies [47,65,66]. These approaches have generated preclinical tools with anti-inflammatory activity in animal models but to date only a monoclonal antibody selective for citrullinated histones (CIT-013) has progressed into clinical development for HS (Figure 3 and [47,66]). First-in-human Ph.1 studies have shown that CIT-013 is well tolerated and bioavailable by the sub-cutaneous route. A multi-center randomized, double-blind, placebo-controlled phase 2 study of CIT-013 in moderate to severe HS patients has been initiated and is anticipated to complete during the first half of 2027 (NCT06993233). In this study, peri-lesional biospies from a subset of patient volunteers will be taken at baseline and at the end of the study to assess translational endpoints including neutrophil infiltration, NET levels and numerous endpoints indicative of the innate and adaptive immune status of the lesion.

### 1.5. Safety Considerations Related to Neutrophil-Targeted Therapies

Support for the premise that neutrophil-mediated inflammation can be reduced while preserving core immune function comes from the genetic disease Papillon-Lefévre syndrome (PLS) and its features. PLS is a rare, autosomal recessive genetic disease caused by loss-of-function mutations in the DPP-1 gene and is characterized by palmoplantar keratosis and severe prepubertal periodontitis. FLS patient-derived neutrophils are also significantly impaired in their ability to form NETs [67]. Despite a phenotype with loss of NSP function and defective NET formation, FLS sufferers are not systemically immunocompromised. Consistent with this, 52 weeks of brensocatib exposure in bronchiectasis patients showed no observed increase in the incidence of bacterial infections or gingivitis [42]. Furthermore, during 20 weeks of exposure to eltrekibart, HS patients showed only mild to moderate adverse events at similar rates to placebo [37] and finally, in healthy volunteers and RA patients, exposure to CIT-013 following subcutaneous administration was well tolerated.

### 1.6. Additional Therapeutic Approaches in HS

The approval of targeted therapies against TNF-α, IL-17A and both lL-17A and IL-17F for moderate-to-severe HS (in 2015, 2023 and 2024 respectively) has shifted treatment from reliance on antibiotics and surgery toward long-term biologic therapy with improved patient outcomes. Furthermore, improving standards of care and reduced patient stigma have enabled earlier therapeutic intervention and brought to light a significant underestimation of the HS patient population. However, improved understanding of disease heterogeneity and modest benefits with current standards of care have focused attention on the need for new therapeutic modalities targeting broader acting, biologically distinct pathways in HS.

While this article has focused on approaches in clinical development that directly target the neutrophil, it is important to acknowledge that additional therapeutic programs are also advancing in HS. Table 1 summarizes these approaches and their stage of clinical development at the time of writing.

Whether any one individual approach will be capable of a step change in symptom improvement or enable prolonged remission remains to be established. While an expanded array of effective and mechanistically distinct therapies for HS is a possibility in the near future, maximizing disease control across a wider patient population may depend on combining well-tolerated agents that engage complementary, orthogonal pathways. Neutrophil targeting strategies are a differentiated and broad acting therapeutic approach with which combinations may be evaluated in the future.

### 1.7. Concluding Remarks

Current immunomodulatory strategies for HS and other neutrophilic inflammatory disorders have largely repurposed biologics such as IL-17 inhibitors, that target adaptive immunity. Despite neutrophils being among the most abundant cell type in HS lesions, they remain an underexplored therapeutic target partly due to limited understanding of their complex biology compared with other immune components. Novel approaches that selectively target neutrophil functions such as chemotaxis, neutrophil serine protease activity and NET formation, offer the potential to reduce HS-related inflammation while preserving broader immune competence. Given the central role of neutrophils in pus formation, such strategies may also alleviate the pain, drainage and odor which constitute a major component of the symptom burden for individuals living with HS.

## Figures and Tables

**Table 1:** Additional therapeutic programs in HS.

Target	Modality	Drug Name	Approved for Use in Other Indications	Stage of HS Development
Janus kinase 1	Small molecule kinase inhibitor	Povorcitinib	Yes	Phase 3
		Upadacitinib	Yes	Phase 3
Bruton’s tyrosine kinase	Small molecule kinase inhibitor	Remibrutinib	Yes	Phase 3
IL-1β	Neutralizing mAb	AVTX-009	No	Phase 2b
IL-1α & IL-1β	Dual targeting, neutralizing mAb	Litikizumab	No	Phase 3
IL-36 receptor	Receptor antagonist mAb	Spesolimab	Yes	Phase 2b/3
IL-17A & IL-17F	Dual targeting, neutralizing nanobody	Sonelokimab	No	Biologics license application in preparation
TL1A	Neutralizing mAb	Tulisokibart	No	Phase 2b
TNF-α & OX40L	Dual targeting, neutralizing mAb	Brivekimeg	No	Phase 2b

## Data Availability

No new data were created or analyzed in this study. Data sharing is not applicable.
